# Circular RNA circXPO1 Promotes Multiple Myeloma Progression by Regulating miR-495-3p/DNA Damage-Induced Transcription 4 Axis

**DOI:** 10.1089/dna.2023.0288

**Published:** 2024-01-12

**Authors:** Fangmei Li, Jing Liu, Jiyu Miao, Fei Hong, Rui Liu, Yang Lv, Yun Yang, Aili He, Jianli Wang

**Affiliations:** Department of Hematology, The Second Affiliated Hospital of Xi'an Jiaotong University, Xi'an City, China.

**Keywords:** circXPO1, miR-495-3p, DNA damage-induced transcription 4, multiple myeloma

## Abstract

Multiple myeloma (MM) is a hematologic malignancy that results from uncontrolled plasma cell proliferation. Circular RNAs are versatile regulators that influence cancer aggression. The pathogenic mechanism of circXPO1 in MM is still unknown. In this study, the expression of circXPO1, miR-495-3p, and DNA damage-induced transcription 4 (*DDIT4*) was detected. Knockdown and overexpression assays were used to evaluate the effect of circXPO1 on MM. Specifically, 5-ethynyl-2′-deoxyuridine and cell counting kit-8 assay were used to investigate cell proliferation. Meanwhile, flow cytometry was adopted to detect cell apoptosis and cell cycle. Apoptosis-associated and cell cycle-related proteins were detected by Western blot. Mechanistically, biotin RNA pull-down assay and dual-luciferase assay were implemented to verify the combination among miR495-3p and circXPO1 or *DDIT4*. The function of circXPO1 *in vivo* was explored in xenograft experiments. The results showed that circXPO1 was up-regulated in both MM samples and MM cell lines and miR-495-3p was down-regulated in MM patients. Silencing circXPO1 inhibited cell proliferation, increased apoptosis rates, and caused the G1 phase arrest. Overexpression of circXPO1 yielded opposite results. In addition, RNA pull-down experiment demonstrated the interaction between circXPO1 and miR-495-3p. Silencing miR-495-3p rescued the inhibitory function caused by the knockdown of circXPO1. *DDIT4* was the target of miR-495-3p. Finally, silencing circXPO1 inhibited the growth of subcutaneous tumors *in vivo*. In conclusion, our findings showed that circXPO1 could promote MM progression via the miR-495-3p/*DDIT4* axis.

## Introduction

Multiple myeloma (MM) is a very common hematological cancer. As of 2021, MM constituted for 1.84% of newly diagnosed cancers and 2.0% of all cancer deaths (SEER, 2021). At present, although our understanding of MM pathological mechanisms and advances in therapeutic approaches continue to improve, there is no curative treatment for MM.

Autologous hematopoietic stem cell transplantation and several novel treatments, such as CAR-T cell therapy, CD138 monoclonal antibody, and XPO1 inhibitors, have achieved significant success (Al Hamed et al., 2019; Cid Ruzafa et al., 2016; Firth, 2019). Even so, a large number of patients finally relapse and develop drug resistance (Gonzalez-Santamarta et al., 2020). Therefore, a deeper understanding of MM pathogenesis is urgently needed.

Covalently closed circular RNAs (circRNAs) are non-coding RNAs that have a covalent closed loop structure and are generated by back-splicing of precursor mRNAs (Li et al., 2020b; Li et al., 2018). CircRNAs play important roles in cancers by regulating cell proliferation (Hsiao et al., 2017), metastasis (Han et al., 2017), cell cycle progression (Du et al., 2016), and apoptosis (Geng et al., 2016). Mechanistically, circRNAs are involved in tumorigenesis by acting as microRNA (miRNA) sponges, binding ribonucleoproteins, and translation templates (Memczak et al., 2013; Weigelt et al., 2020; Xia et al., 2018).

Increasing studies have demonstrated that the dysregulation of circRNAs is associated with multiple tumor progression, such as melanoma, acute myeloid leukemia, hepatocellular carcinoma (Hanniford et al., 2020; Huang et al., 2020a; Liu et al., 2021b), and MM (Perez de Acha et al., 2020). For example, high expression of circ_0001821 predicted a poor prognosis for MM. Circ_0001821 could promote cell proliferation and inhibit cell apoptosis, to promote MM progression (Liu et al., 2021a).

Circ-MYBL2, as a tumor inhibitor, could repress MM cell proliferation through the circ-MYBL2/Cyclin F/*MYBL2* axis (Yu et al., 2021). CircXPO1 is produced from the *XPO1* gene, a well-acknowledged target for cancer therapy (Huang et al., 2020b). CircXPO1 is involved in the progression of many cancers. Overexpression of CircXPO1 could promote the progression of lung adenocarcinoma by stabilizing *CTNNB1* mRNA (Huang et al., 2020b).

CircXPO1 could promote the oncogenesis of prostate cancer by targeting miR-23a (Chen et al., 2021). CircXPO1 could regulate multiple miRNAs and, enhance the proliferation capacity and migration of osteosarcoma cells via up-regulating *XPO1* (Jiang et al., 2020). However, the role of circ-XPO1 in MM remains unknown.

As a member of non-coding RNAs, miRNAs regulate gene expression at the post-transcriptional level (Lu and Rothenberg, 2018). CircRNAs bind to miRNA to reduce the binding of miRNA to downstream target genes (Bushati and Cohen, 2007). For instance, circ-ZEB1 up-regulates *PIK3C* level via binding to miR-199a-3p, influencing the proliferation and apoptosis of hepatocellular carcinoma (Liu et al., 2022). miR-495-3p has been reported to play an important role in cervical cancer (Tang et al., 2021) and colorectal carcinoma (Zhang et al., 2022).

In this study, we demonstrated the high expression of circXPO1 in MM patients and MM cell lines. Further, circXPO1 was shown to promote MM progression via the miR-495-3p/DNA damage-induced transcription 4 (*DDIT4*) axis. These results may provide novel insights into possible treatment approaches for MM.

## Materials and Methods

### Clinical samples and cell culture

Fifty-four newly diagnosed MM patients and 17 healthy controls were enrolled in this study, from the Department of Hematology of the Second Affiliated Hospital between 2015 and 2020. The research was approved by the Ethics Committee of Xi'an Jiaotong University (Approval No. 2015186). The statistical results of 54 patients are shown in Table 1.

**Table 1.** Relationship Between circXPO1 Expression and Clinicopathologic Features in 54 Patients with Multiple Myeloma

Characteristics	Number (*n* = 54)	Relative expression of circXPO1, mean ± SD	*p*
Age, years			0.867
≤60	25	4.52 ± 2.13	
>60	29	4.55 ± 1.32	
Gender			0.551
Male	31	4.83 ± 1.65	
Female	23	4.21 ± 3.22	
M Protein			0.255
IgG	29	4.35 ± 1.13	
IgA	14	4.12 ± 1.44	
IgD	4	4.88 ± 1.83	
Light chain	11	4.27 ± 1.65	
β_2_-MG, mg/L			*0.003*
<5.5	33	5.56 ± 1.85	
>5.5	21	2.87 ± 1.67	
Percentage of myeloma cells in BM			0.277
<30%^a^	16	4.31 ± 1.33	
>30%	38	4.75 ± 0.54	
ISS stage			*0.0024*
I	23	3.65 ± 0.52	
II	22	4.92 ± 0.48	
III	9	5.98 ± 0.23	
D-S stage			0.395
I	13	4.10 ± 1.12	
II	30	4.31 ± 0.85	
III	11	6.21 ± 0.98	
LDH, U/L			0.333
>240	37	4.74 ± 1.37	
<240	17	3.95 ± 1.99	
Bone disease			0.215
With	38	4.69 ± 1.23	
Without	16	4.12 ± 1.58	

Italic values represent statistically significant differences.

^a^ Thirty percent is the mean value of the percentage of myeloma cells in BM of 54 MM patients.

BM, bone marrow; ISS, International Staging System; D-S, Durie-Salmon; LDH, lactate dehydrogenase; MM, multiple myeloma; SD, standard deviation.

Human MM cell lines (MM.1S, OPM2, U266, NCI-H929, RPMI-8226) and human bone marrow stroma cell (HS-5) were all acquired from the Cell Bank of Chinese Academy of Science and cultivated in RPMI-1640 medium or Dulbecco's modified Eagle's medium (Invitrogen) added with 10% fetal bovine serum at 37°C. All *in vitro* studies were performed repeatedly three times in cell lines.

### CircRNA microarray

A circRNA microarray included five MM patients, and five healthy controls provided by KangChen Biotech (Shanghai, China) were used in this study. The microarray data generated in this study are available in the Gene Expression Omnibus database repository (www.ncbi.nlm.nih.gov/geo, Accession No. GSE208782). Specifically, mononuclear cells were extracted from donor bone marrow by Ficoll density gradient centrifugation and then total RNA was extracted by Trizol.

Total RNA from each sample was quantified using the NanoDrop ND-1000. The sample preparation and microarray hybridization were performed based on the Arraystar's standard protocols. Total RNA was digested with Rnase R (Epicentre, Inc.) to remove linear RNAs and enrich circRNAs. Then, the enriched circRNAs were amplified and transcribed into fluorescent complementary RNA (cRNA) using a random priming method (Arraystar Super RNA Labeling Kit; Arraystar). The labeled cRNAs were hybridized onto the Arraystar Human circRNA array. After having washed the slides, the arrays were scanned by the Aligent Scanner G2505C.

Agilent Feature Extraction software (version 11.0.1.1) was applied to analyze obtained array images. Quantile normalization and subsequent data processing were performed using the R software limma package (Ritchie et al., 2015). Differentially expressed circRNAs (DECs) with statistical significance (*p* ≤ 0.05) between two groups were identified through Volcano Plot filtering. The DECs between two samples were identified through fold change (FC) filtering (FC ≥2). Hierarchical clustering was performed to show the distinguishable circRNAs expression pattern among samples.

### RNA stability assay

MM cells were treated with 1 μg/mL actinomycin D (Sigma) for 0, 4, 8, 12, and 24 h to block transcription. Next, we collected the cells and extracted total RNA; then, we assessed the abundance of circXPO1 and XPO1 mRNAs by quantitative real-time PCR (qRT-PCR). The stability of the circRNA was determined as described in previous publications (Ritchie et al., 2015).

### RNase R assay

Total RNA was extracted from MM cells; then, RNase-free water (Mock) or RNase (Lucigen) was added and incubated at 37°C for 30 min. Treated RNA was refined by the RNeasy MiniElute Cleanup kit (Qiagen) according to the manufacturer's instructions. The concentration-refined RNA was determined, and then RNA with a total amount of 1 μg was applied for qRT-PCR detection to assess the abundance of circXPO1 and linear *XPO1*. The Rnase R assay was determined as described in previous publications (Li et al., 2020a; Mfossa et al., 2019).

### Nuclear and cytoplasmic fractionation

Nucleocytoplasmic separation experiment was performed with NE-PER kit (Thermo Scientific). According to the specification, the RNA of nucleus and cytoplasm were obtained by centrifugation after cell lysis with lysate. The concentration of the extracted RNA was determined using a NANO-Drop spectrophotometer. Then, the expression levels of U6 and β-actin were measured by qRT-PCR and used to determine the effectiveness of nucleoplasmic separation.

### Cell transfection

Small interfering RNA (siRNA) or the negative control RNA (siRNA-NC) and miR-495-3p mimics (miR-495-3p) or its inhibitor (inhibitor-miR-495-3p) and negative control (miR-NC) were synthesized by RiboBio (Shanghai, China). One hundred nanomoles miRNA or siRNA were transfected into MM cells by RFectsp siRNA/miRNA Transfection Reagent (Baidai biotechnology, Changzhou, China). The plasmid for circXPO1 overexpression was designed based on pEX-3, which was produced by GenePharma (Shanghai, China).

The plasmids were delivered into cultured MM cells by LipoFiter (Hanbio, Shanghai, China). MM cells (3 × 10^5^ cells/sample) were transfected with 100 nmol siRNA, miRNA or 2.5 μg plasmids for 72 h. Transfection efficiency was measured by qRT-PCR.

Lentiviruses containing circXPO1 short hairpin RNA (sh-RNA) were used to transfect MM.1S cells to establish a stable circXPO1 knockdown MM cell line for xenograft assay. Sh-circXPO1 and negative control (sh-NC) lentivirus were purchased from GenePharma.

The sequence of shRNA is as follows: sh-circXPO1: 5′-CCATGTTATTCAAGATGCTTC-3′, sh-NC: 5′-GTTCTCCGAACGTGTCACGT-3′. For lentivirus transfection, the MM.1S cells were added with lentivirus (multiplicity of infection = 100:1) and 7 μg/mL polybrane, then centrifuged at 200 *g* rotational speed for 1.5 h and continued to culture for 72 h, and then used the culture medium containing 1 μg/mL perinomycin (Thermo Fisher Scientific, Shanghai, China) to obtain the circXPO1 stable knockdown MM cell line.

### Cell counting kit-8 assay

Cell counting kit-8 (CCK-8) assays were conducted to measure the viability of cells. Transfected MM cells (8 × 10^3^/well/100 μL) were added into 96-well plates and cultured for 0, 24, 48, 72, and 96 h; then, 10 μL CCK-8 solution per well was added (7Sea Pharmatech, China), followed by a 4-h incubation. The absorbance was measured at 450 nm by a microplate reader.

### Cell apoptosis and cell cycle detection

The apoptosis and cell cycle were detected by flow cytometry. Transfected MM cells were cultured for 72 h. Then, the cells were collected to determine the proliferation and apoptosis rates. During cell cycle analysis, the collected cells were treated with 70% ice ethanol at 4°C for 12 h; then, 10 μL PI solution (Beyotime, Shanghai, China) and 5 μL Rnase A (Beyotime) per sample were added and incubated for 30 min based on the relevant instructions.

Apoptosis rates were detected by Annexin V-PE/RedNucleus II kit (Bioscience). The transfected MM cells were collected, 5 μL PE reagent and 5 μL RedNucleus II were added to each sample, and they were incubated for 15 min at room temperature. All flow cytometry tests were performed by flow cytometry NovoCyte (ACEA Bioscience), and the experimental data were processed by Novo software (ACEA Bioscience).

### 5-Ethynyl-2′-deoxyuridine staining assay

Cell proliferation was evaluated using 5-ethynyl-2′-deoxyuridine (EdU) assay kit (Beyotime). After transfection for 48 h, cells were (2 × 10^5^ cells/plate) incubated with EdU reagent for 2 h. After that, cells were collected, fixed by 4% paraformaldehyde (Biosharp, Shanghai, China), and permeated with Triton X-100 solution (Beyotime). Finally, a click reaction solution containing Azide 647 was added to stain cells at room temperature for 30 min. EdU-positive cells were measured via flow cytometry NovoCyte (ACEA Bioscience).

### Chromatin isolation by RNA purification

This assay was done with reference to previously published studies (Chu et al., 2012). The circRNA chromatin isolation by RNA purification (ChiRP) probe (biotin-labeled circXPO1 probe) and corresponding NC were purchased from RiboBio (Guangzhou, China).

The prepared MM cells (20 million per sample) were cross-linked, lysated, and sonicated with the cell lysate; then, biotin-labeled circXPO1 ChiRP was added to the probe, hybridized with chromatin and Streptavidin-coupled magnetic C1 beads (Invitrogen) were used to collect chromatin; and RNA was subsequently isolated from ChiRP samples for quantification by qRT-PCR.

### Luciferase reporter assay

In the process of bioinformatics analysis, StarBase v2.0 (Li et al., 2014) and Circular RNA Interactome software (Dudekula et al., 2016) were selected. Wild-type (WT) and mutant reporter plasmids of circXPO1 or *DDIT4* were synthesized and cloned into pmirGLO vectors (Promega) according to relevant experimental requirements. On this basis, MM cells were transfected with plasmids and miRNA mimics/NC for 48 h. The corresponding luciferase activity was tested by the Dual-luciferase reporter assay kit (Promega), and the relative activity results are obtained based on the comparison.

### Quantitative RT-PCR

Total RNA was isolated using Trizol reagent (Sigma Aldrich), RNA concentration was measured by a NanoDrop spectrophotometer (Thermo Fisher Scientific), and then cDNA was synthesized using HiFi Script cDNA Synthesis Kit (Cowin Biotech, Beijing, China) or miRcute Plus miRNA First-Strand cDNA Kit (Tiangen Biotech, Beijing, China).

The qRT-PCR was conducted by Ultra SYBR Mixture (Cowin Biotech). β-Actin (for circRNA) and U6 (for miRNA) were internal controls. Relevant primers involved in the current experiments were designed from Tsingke Biotechnology (Beijing, China), and the sequences of primers are shown in Table 2.

**Table 2.** Primer Sequence

RNAs	Primer sequences
β-Actin	Forward: 5′-GTGGCCGAGGACTTTGATTG-3′
	Reverse: 5′-CCTGTAACAACGCATCTCATATT-3′
U6	Forward: 5′-CTCGCTTCGGCAGCACA-3′
	Reverse: 5′-AACGCTTCACGAATTTGCGT-3′
hsa_circRNA_102735	Forward: 5′-ATCATTTGGCTGCTGAACTCTA-3′
	Reverse: 5′-AGTTCTGTTCATCATCTTTTCCAT-3′
hsa_circRNA_105013	Forward: 5′-TTCTTCCAGGTGATGGTGAGGT-3′
	Reverse: 5′-CTTGGTCATTGTGTGTGC-3′
hsa_circRNA_104423	Forward: 5′-CCACTGGCAAAGAGTCACCTAAA-3′
	Reverse: 5′-ATTCCCTGGCAGTTCCGTGTA-3′
DDIT4	Forward: 5′-TGCATTGGGGACACATACCC-3′
	Reverse: 5′-CCCAAGTGATCCCTGACACC-3′

### Western blot

Total protein was extracted with RIPA lysis buffer (Xianfeng Biotechnology, Xi'an, China), and quantitative analysis was conducted through the BCA Protein Assay Kit (Beyotime). Forty micrograms protein samples were loaded on 10% sodium dodecyl sulfate–polyacrylamide gel electrophoresis (SDS-PAGE) and transferred into polyvinylidene fluoride membrane (Millipore); then, they were sealed with 5% defatted milk for 1 h.

After sufficient cleaning, they were incubated with primary antibodies: DDIT4 (1:1000; Proteintech), P21 (1:2000; Proteintech), Bcl2 (1:2000; Proteintech), BAX (1:8000; Proteintech), cMYC (1:2000; Proteintech), CDK2 (1:2000; Proteintech), or β-tubulin (1:5000; Proteintech) at 4°C for 16 h. Subsequently, secondary antibodies (1:10,000; Proteintech) were incubated for 1 h. Protein signals were then visualized with ECL reagents (Beyotime).

### MM mouse xenograft model

Experimentation on animals was implemented by strictly following the ARRIVE guidelines (Kilkenny et al., 2010). The animal experiments were approved by the Medical Biology Research Ethics Committee (Approval No. 2022-1498). Ten 4-week-old male BALB/cNj-Foxn1^nu^/Gpt nude mice (GemPharmatech, China) were used in animal experiments.

The experiment was repeated three times. A total of 1 × 10^7^ MM.1S-sh-circXPO1/NC cells in 50 μL phosphate-buffered saline together with 50 μL Matrigel matrix (Corning) was injected in the inguinal area of mice to construct the MM xenograft model. The experimental animals were bred in the Animal Experimental Center of Xi'an Jiaotong University in an SPF-level environment.

The two orthogonal diameters (*a* and *b*) were detected every 2 days, and xenograft volume was calculated based on *V* = *ab*^2^/2. The experimental animals were killed by cervical dislocation after intraperitoneal injection of pentobarbital sodium at 28 days post-injection. Xenograft was paraffin-embedded for hematoxylin and eosin staining and immunohistochemistry (IHC) analysis.

### Statistical analysis

SPSS STATISTICS 18.0 (SPSS, Inc., Chicago, IL) software tools were used in data analysis. The measurement results obtained from the experiment were described by mean ± standard deviation, and Student's *t-*test was conducted to confirm whether there is a statistical difference between groups. The correlation analysis was judged by Spearman's correlation analysis. Associations between circXPO1 expression and clinic pathological features were analyzed based on the Mann–Whitney *U* test. Statistical significance was judged by *p* < 0.05.

## Results

### The expression of circXPO1 and its role in prognosis for MM

**FIG. 1.**
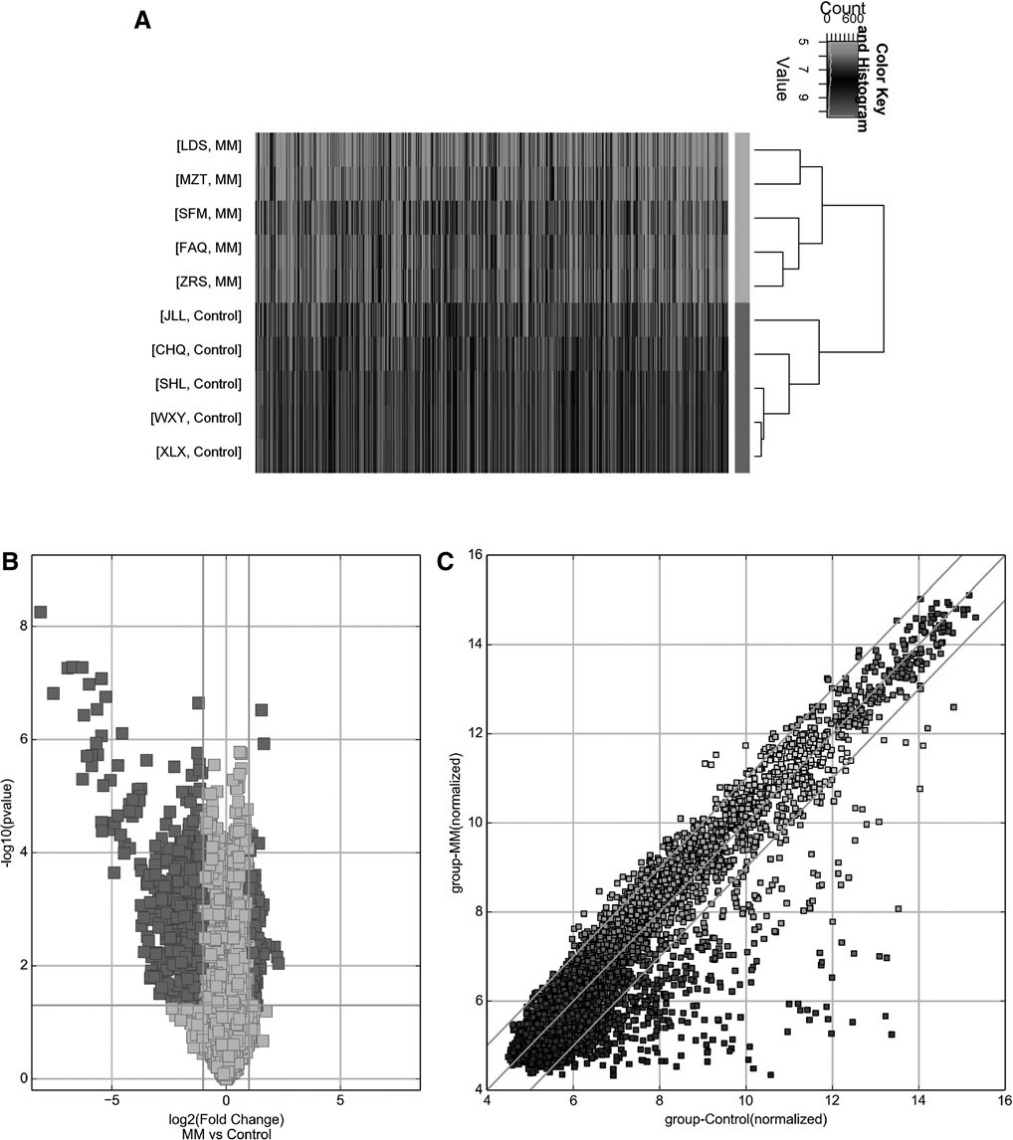
Differentially expressed circRNA in MM. **(A)** Clustering heat map of circRNAs in MM (*p* < 0.05). **(B)** Volcanic plot reflecting circRNAs with ≥2-fold changes in BM of the two groups from MM patients (*n* = 5) or healthy controls (*n* = 5). **(C)** Differences in circRNA expression described by scatter plot. BM, bone marrow; circRNA, circular RNA; MM, multiple myeloma.

The circRNA expression profile of newly diagnosed MM was evaluated by a circRNA microarray analysis with five newly diagnosed MM patients and five healthy controls. Clustering heat maps were drawn to assess DEC in the two groups (Fig. 1A). A total of 577 circRNAs was differentially expressed (*p* < 0.05 and FC ≥2.0), with 440 down-regulated circRNAs and 137 up-regulated circRNAs in newly diagnosed MM patients (Fig. 1B, C).

We selected a number of star miRNA molecules from the published literature that have been widely validated and play a key role in initial MM as a bridge to screen key circRNAs in MM. Based on the results of miRNA target prediction, we screened out some relevant circRNAs (Supplementary Table S1). We selected three circRNAs (hsa_circRNA_105013, hsa_circRNA_104423, and hsa_circRNA_102735) according to *p* value, FC, and original value, and we further verified these differential expression circRNAs in MM cell lines and healthy controls by qRT-PCR.

**FIG. 2.**
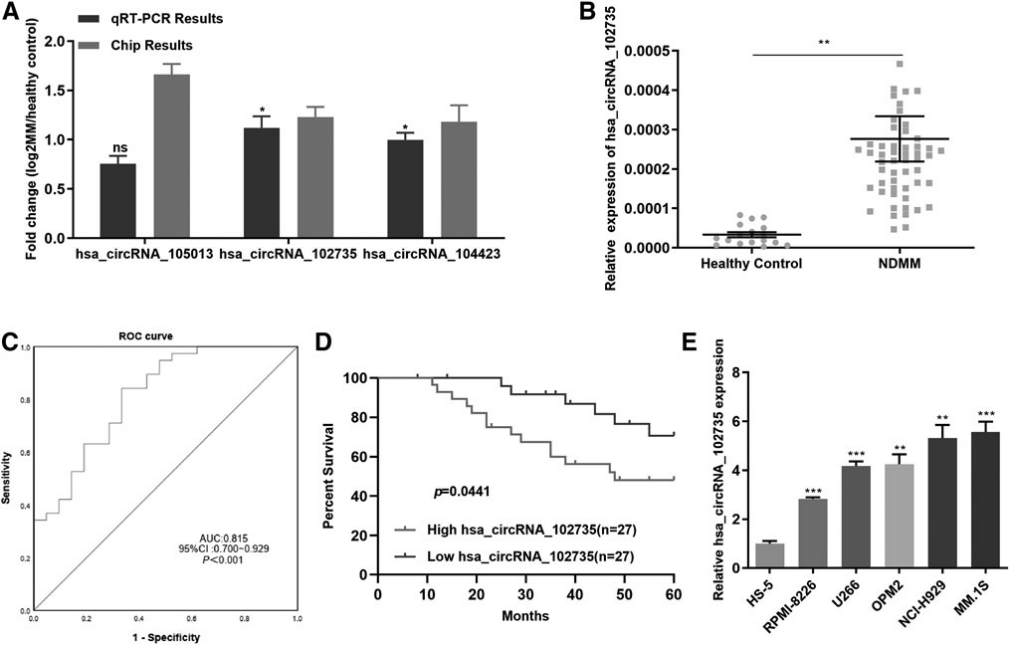
**(A)** Expression of three circRNAs in patients and control samples as measured by qRT-PCR and by chip microarray. **(B)** BM circXPO1 expression in newly diagnosed MM patients (*n* = 54) and healthy controls (*n* = 17) was texted by qRT-PCR. **(C)** ROC curve and AUC values. **(D)** Overall survival in MM patients with high circXPO1 expression versus low circXPO1 expression. **(E)** The expression of circXPO1 in five MM cell lines and stromal cells HS-5 was detected by qRT-PCR. *p < 0.05, **p < 0.01, ***p < 0.001. qRT-PCR, quantitative real-time PCR; ROC, receiver operating characteristic curve.

qRT-PCR results showed that hsa_circRNA_105013 was highly expressed in MM cell lines compared with healthy controls, but the difference was not statistically significant (*p* = 0.4918). We chose hsa_circRNA_102735 with large FC as the further research target (Fig. 2A).

Based on circBase (www.circbase.org/) annotation, hsa_circRNA_102735 was from the *XPO1* (Exportin 1) gene at chr2:61721028-61722748 and named as circXPO1. We detected circXPO1 expression in BM samples of 17 healthy controls and 54 newly diagnosed MM patients.

We found that the level of circXPO1 presented high expression in newly diagnosed MM patients than in healthy controls, consistent with the results of the chip (Fig. 2B). The clinical information of the 54 newly diagnosed MM patients was presented in Table 1. We found that circXPO1 levels were positively related to blood β_2_-MG levels and ISS stage (Table 1, *p* < 0.05).

The receiver operating characteristic (ROC) curves were applied to assess the sensitivity and specificity of circXPO1 for MM diagnosis. Comparisons were made of circXPO1 levels in BM of newly diagnosed MM patients and healthy controls. The area under the curve of circXPO1 was 0.815 (Fig. 2C). Survival analysis revealed that patients with high circXPO1 expression had a poor survival (Fig. 2D).

We used qRT-PCR to detect circXPO1 expression in MM cell lines. CircXPO1 expression was enhanced in MM cells (OPM2, MM.1S, U266, RPMI-8226, and NCI-H929) relative to the marrow stromal cell line, HS-5 (Fig. 2E). CircXPO1 expression was greatest in MM.1S and NCI-H929 compared with the other MM cell lines and HS-5. Those results indicated that circXPO1 was high in MM patients and cells, and it was related with poor prognosis.

### Identification and validation of circXPO1 in MM

**FIG. 3.**
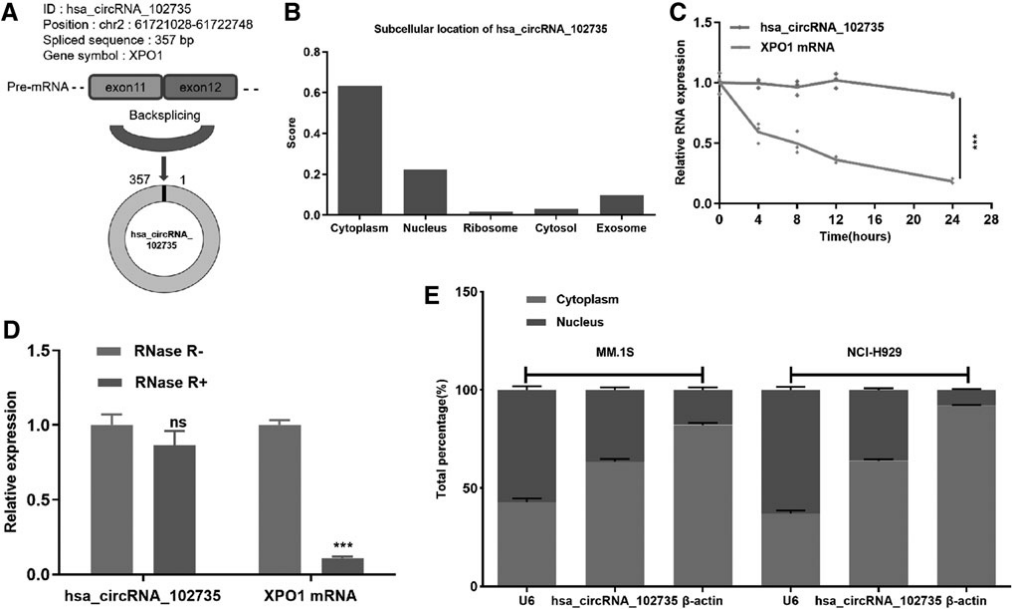
Identification of circXPO1 in MM. **(A)** Schematic diagram of exonic regions of circXPO1 (hsa_circRNA_102735). **(B)** CircXPO1 is located in the cytoplasm as predicted by IncLocator. **(C)** Time-course of relative circXPO1 and XPO1 expression in MM.1S cells at different time points. **(D)** RNA samples treated with or without RNase R. qRT-PCR detected the expression of XPO1 and circXPO1. **(E)** CircXPO1 was mostly located in the cytoplasm. ***p < 0.001.

The presence of circXPO1 was verified in the circBase database and the circBank database. Genomic structure demonstrated that circXPO1 is located at 2p and contains a relatively large second exon (357 bp) generated by back-splicing of the XPO1 gene (Fig. 3A). CircXPO1 potential subcellular location was predicted by the online tool, IncLocator (www.csbio.sjtu.edu.cn/bioinf/lncLocator/).

As shown in Figure 3B, circXPO1 was primarily located within the cytoplasm with a predictive score of 0.62. To further characterize circXPO1, the stability and location of circXPO1 in MM cells were analyzed. After the cells were cultured with medium containing actinomycin D, results showed that the half-time of circXPO1 was significantly long compared with XPO1 mRNA, indicating the stability of circXPO1 (Fig. 3C). In addition, compared with linear *XPO1*, circXPO1 could resist RNase R, indicating that circXPO1 was a stable circRNA in MM cells (Fig. 3D). Further, qRT-PCR analysis demonstrated that circXPO1 was preferentially localized in the cytoplasm, with a moderate localization within the nucleus (Fig. 3E).

### CircXPO1 regulates the proliferation and cell cycle progression of MM cells

**FIG. 4.**
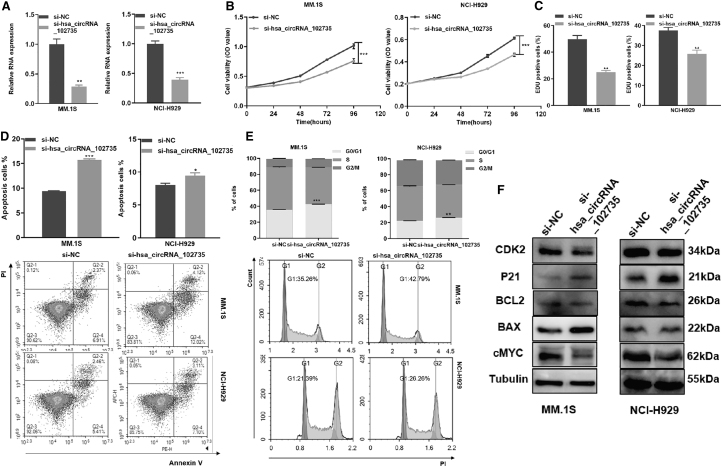
Knockdown of circXPO1 promoted proliferation, cell cycle progression and decreased apoptosis**. (A)** The efficiency of circXPO1 silence in MM.1S and NCI-H929 cell lines. **(B)** Cell viability of MM cells after circXPO1 knockdown was tested by CCK-8 assay. **(C)** Cell proliferation capacity was evaluated by EdU assay. **(D)** Apoptosis of MM cells after circXPO1 knockdown for 48 h. **(E)** Cell cycle profiles of MM cells transfected with circXPO1 siRNA. **(F)** WB measured the cell cycle- and apoptosis-associated molecules. *p < 0.05, **p < 0.01, ***p < 0.001. CCK-8, cell counting kit-8; EdU, 5-ethynyl-2′-deoxyuridine; siRNA, small interfering RNA.

siRNA was used to knockdown circXPO1 expression in MM cell lines. qRT-PCR confirmed the knockdown efficiency (Fig. 4A). By the CCK-8 assay, knockdown of circXPO1 significantly decreased MM cell viability (Fig. 4B). EdU staining assay confirmed that the knockdown of circXPO1 inhibited cell proliferation (Fig. 4C).

Knockdown of circXPO1 statistically increased the apoptotic rate of MM cells (Fig. 4D). By flow cytometry, circXPO1 knockdown caused cell cycle arrest at G0/G1 phase (Fig. 4E). Moreover, specific cyclins Cdk2 and p21 in the G1/S phase, apoptosis associated proteins Bcl-2, bax, and proliferation-related proteins c-Myc were detected by Western blotting. Compared with NC, the si-circXPO1 cells expressed less Cdk2, Bcl-2, and c-Myc proteins but more p21 and Bax proteins (Fig. 4F and Supplementary Data).

**FIG. 5.**
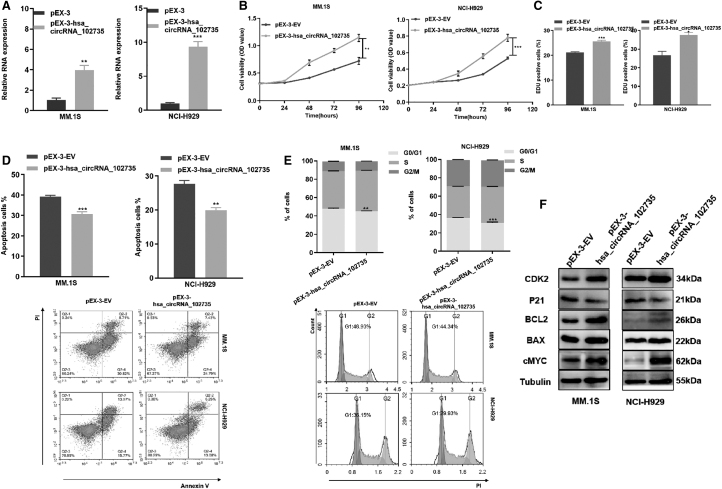
Overexpression of circXPO1 inhibited proliferation ability along with increased apoptosis rates and cell cycle arrest. **(A)** The efficiency of circXPO1 overexpression in MM.1S and NCI-H929. **(B)** Cell viability was assessed by CCK-8 assay. **(C)** Cell proliferation was assessd by EdU assay. **(D)** Apoptosis of MM cells was assessed by flow cytometry after transfection with pEX-3-EV or pEX-3-circXPO1 for 48 h. **(E)** Cell cycle profiles of MM cells transfected with pEX-3-EV or pEX-3-circXPO1. **(F)** WB measured the cell cycle- and apoptosis-associated factors. *p < 0.05, **p < 0.01, ***p < 0.001.

pEX-circXPO1 was used to overexpress circXPO1, and qRT-PCR confirmed the overexpression efficiency (Fig. 5A). Gain-of-function studies showed that up-regulated circXPO1 promoted MM cell proliferation and G1-S transition (Fig. 5B–E). In addition, the expression levels of Cdk2, Bcl-2, and c-Myc were increased, whereas p21 and Bax were decreased in MM cells transfected with pEX-circXPO1 (Fig. 4F and Supplementary Data).

### CircXPO1 directly interacts with miR-495-3p

**FIG. 6.**
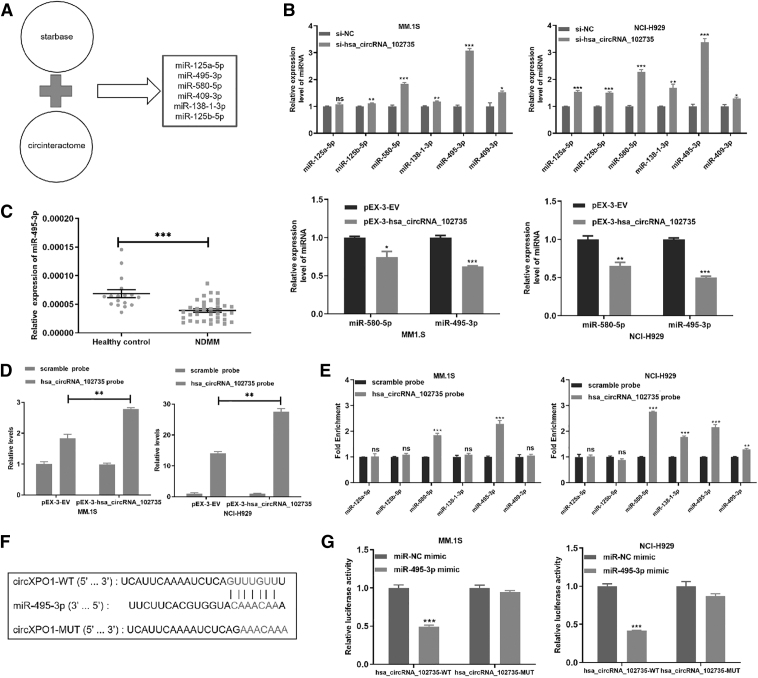
CircXPO1 can bind to miR-495-3p. **(A)** Six candidate miRNAs that bind the 3′-UTR of circXPO1 were predicted by online bioinformatics network. **(B)** qRT-PCR measured the level of candidate miRNAs after circXPO1 knockdown or overexpression. **(C)** The expression of miR-495-3p in healthy control and NDMM patients was detected by qRT-PCR. **(D)** Biotin-labeled circXPO1 probe enriched circXPO1 in MM cells. **(E)** Six miRNAs were pulled down and measured by qRT-PCR. **(F)** Predicted potential binding sites of circXPO1 to miR-495-3p. **(G)** Relative luciferase activity of MM cells post-transfection with circXPO1-WT/MUT and miR-495-3p mimics/NC. *p < 0.05, **p < 0.01, ***p < 0.001. miRNA, microRNA; NC, negative control; WT, wild-type.

Endogenous circRNAs often perform functions by interacting with miRNAs. Because circXPO1 predominantly existed in the cytoplasm, we hypothesized that it could sponge miRNAs, affecting downstream gene expression. Through computational prediction, we identified six candidates (miR-125a-5p, miR-125b-5p, miR-409-3p, miR-495-3p, miR-138-1-3p, and miR-580-5p) based on circular RNA CircInteractome (https://circinteractome.nia.nih.gov/) and starbase (http://starbase.sysu.edu.cn/) analysis (Fig. 6A). Then, we detected the expression changes of six candidate miRNAs after circXPO1 knockdown or overexpression.

As expected, circXPO1 knockdown increased miR-495-3p levels, whereas circXPO1 overexpression decreased miR-495-3p expression (Fig. 6B). Next, we measured miR-495-3p expression within the MM patients and healthy controls, and results showed that miR-495-3p was down-regulated within the NDMM samples (Fig. 6C).

To further prove that miR-495-3p was the target of circXPO1, RNA pull-down experiment was performed, and the circXPO1-specific probe was used to enrich circXPO1. Biotin-labeled circXPO1 probe enriched circXPO1 in MM cells (Fig. 6D). The circXPO1 probe was found to significantly enrich miR-495-3p in both MM.1S and NCI-H929 cell lines (Fig. 6E).

In addition, we established a luciferase reporter vector actuated by the WT 3′-UTR sequence of circXPO1. The vector contained a potential miR-495-3p binding site (circXPO1-WT) and a mutated binding site (circXPO1-MUT) (Fig. 6F). The dual-luciferase reporter experiments results showed that luciferase activity decreased significantly after transfection of miR-495-3p in the circXPO1-WT group (Fig. 6G). These results demonstrated that circXPO1 can bind to miR-495-3p.

### CircXPO1 promoted cell proliferation and cell cycle progression by sponging miR-495-3p

**FIG. 7.**
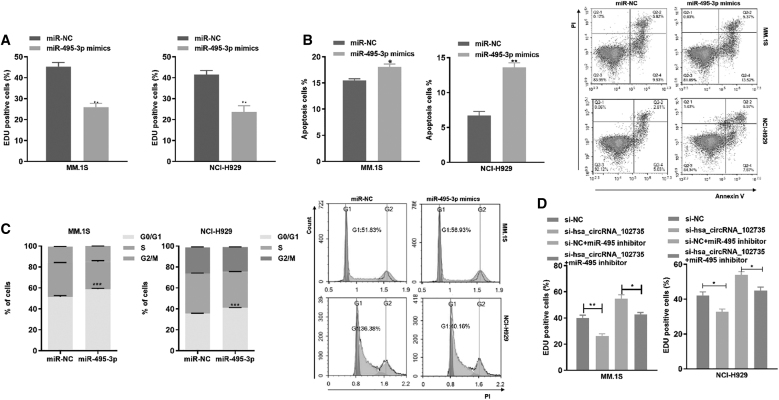

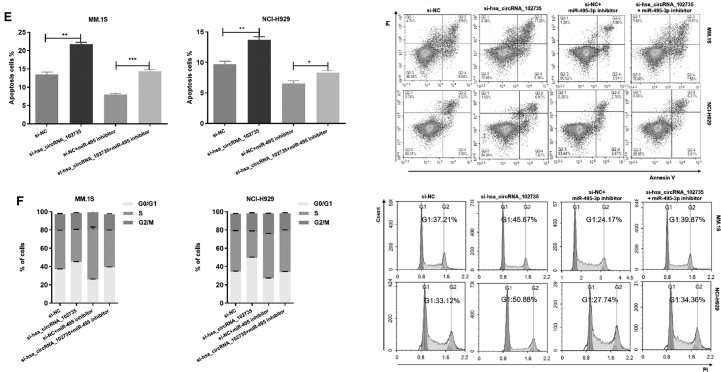
miR-495-3p partially rescues the circXPO1 function. **(A)** The proliferation of MM cells after transfection with miR-NC and miR-495-3p mimics was measured by EdU assay. **(B)** Cells were transfected with miR-495-3p mimics/NC for 72 h, and flow cytometry was applied to determine the apoptotic ratio. **(C)** Cell cycle profile of MM cells transfected with miR-NC or miR-495-3p. **(D)** Cell proliferation analysis of MM cells after down-regulation of circXPO1 and transfection with the miR-495-3p inhibitor. **(E)** Apoptotic changes in MM cells after decreasing circXPO1 expression and transfecting with the miR-495-3p inhibitor. **(F)** Flow cytometry assessment of cell cycle after down-regulation of circXPO1 and treatment with the miR-495-3p inhibitor. *p < 0.05, **p < 0.01, ***p < 0.001.

Previously, circXPO1 was shown to negatively regulate miR-495-3p. We assessed whether circXPO1 contributes to the malignant progression of MM cells by binding to miR-495-3p and found that the miR-495-3p mimic reduced proliferation of MM.1S and NCI-H929 cells (Fig. 7A). Moreover, the miR-495-3p mimic increased the apoptotic rate of MM cells (Fig. 7B) and arrested cell cycle in the G0/G1 phase (Fig. 7C). These results demonstrated that miR-495-3p inhibits MM cell growth and affects cell cycle progression.

To assess whether circXPO1 promoted cell proliferation by inhibition of miR-495-3p, we co-transfected si-circXPO1 and miR-495-3p inhibitor into MM cells. The inhibitor reversed the si-circXPO1-mediated reduction in cell proliferation, decreased the apoptotic ratio, and promoted G1/S phase transition (Fig. 7D–F). These results demonstrated that circXPO1 could promote MM cell proliferation ability, reduce apoptosis, and accelerate cell cycle progression by regulating miR-495-3p.

### DDIT4 is the target gene of miR-495-3p

Bioinformatic approaches used to explore the targets of miR-495-3p, Target Scan (https://www.targetscan.org) (Grimson et al., 2007), miRDB (https://www.mirdb.org) (Chen and Wang, 2020), and microT-CDS (Diana Labs) (Paraskevopoulou et al., 2013) were used to predict the downstream genes of miR-495-3p.

**FIG. 8.**
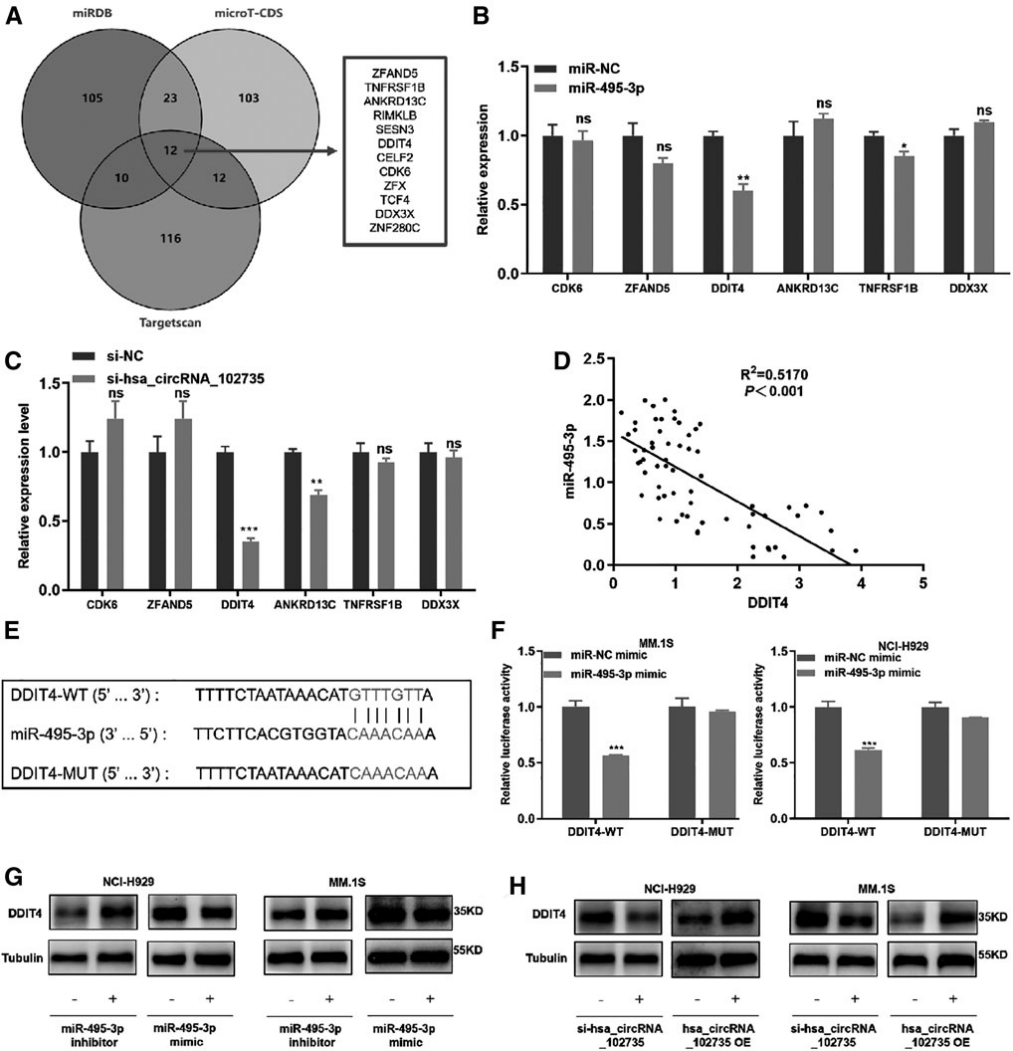
DDIT4 can bind to miR-495-3p. **(A)** The overlap mRNAs predicted by TargetScan, miRDB, and microT-CDS. **(B)** The expression of candidate genes was detected after transfection of miR-495-3p in MM cells. **(C)** Expression levels of downstream candidate target genes were detected after transfection with circXPO1 siRNAs. **(D)** Correlation analysis of DDIT4 and miR-495-3p in MM patients. **(E)** The sequence of miR-495-3p and DDIT4 binding sites and mutation sites. **(F)** After miR-495-3p (or NC) and DDIT4-MUT (or WT) were transfected simultaneously, relative luciferase activity was determined. **(G)** Protein levels of DDIT4 in MM cells after transfection with the miR-495-3p mimic/NC or the miR-495-3p inhibitor/NC measured by Western blot. **(H)** DDIT4 protein levels in MM cells transfected with si-circXPO1/si-NC or circXPO1 OE/NC were assessed by Western blot. *p < 0.05, **p < 0.01, ***p < 0.001. DDIT4, DNA damage-induced transcription 4; OE, overexpression.

The intersection of the top 150 predicted target genes of each databases was taken, and 12 common genes were identified: *ZFAND5, TNFRSF1B, ANKRD13C, RIMKLB, SESN3, DDIT4, CELF2, CD-K6, ZFX, TCF4, DDX3X,* and *ZNF280C* (Fig. 8A). We selected six genes (*ZFAND5, TNFRSF1B, ANKRD13C, DDIT4, CDK6*, and *DDX3X*) that have been studied in tumors for further screening.

We enhanced the level of miR-495-3p with mimics and found that miR-495-3p overexpression significantly decreased the level of *DDIT4* (Fig. 8B) (Sofer et al., 2005). Moreover, *DDIT4* expression was reduced with circXPO1 knockdown (Fig. 8C). We also found that miR-495-3p was a negative correlation with *DDIT4* in MM patients (Fig. 8D).

To assess the relationship between miR-495-3p and *DDIT4*, a luciferase reporter vector was established that contained a DDIT4-WT and a DDIT4-MUT sequence (Fig. 8E). MM.1S and NCI-H929 cells were transfected with DDIT4-WT/MUT plasmids and miR-495-3p mimics/NC, and a dual luciferase assay was conducted to analyze fluorescence levels. With overexpression of miR-495-3p, the luciferase activity of the vector containing DDIT4-WT was significantly decreased, whereas the activity of the vector including DDIT4-MUT was unchanged (Fig. 8F).

Then, the miR-495-3p mimics and corresponding inhibitor were transfected into MM.1S and NCI-H929 cells, and DDIT4 protein expression was assessed by Western blot. When miR-495-3p was overexpressed, the expression of DDIT4 was significantly decreased. Down-regulation or up-regulation of circXPO1 exerted opposite effects. DDIT4 expression decreased after circXPO1 down-regulation and increased after circXPO1 up-regulation (Fig. 8G, H and Supplementary Data). These results confirmed that *DDIT4* was a target of miR-495-3p.

### Down-regulation of circXPO1 inhibits tumor growth *in vivo*

**FIG. 9.**
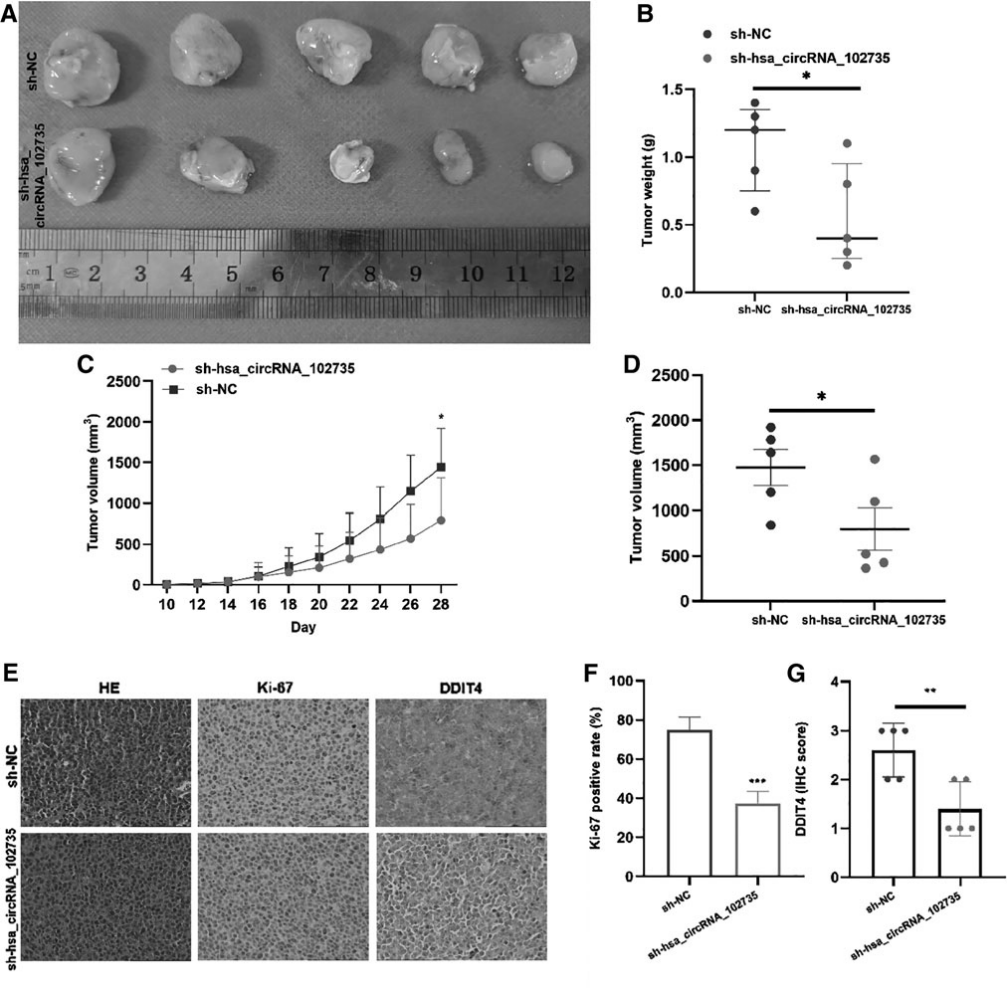
The influence of circXPO1 on MM.1S growth in xenografted nude mice. **(A)** Tumor resection image of nude mice at 28 days after the initiation of the experiment. **(B)** Comparisons of tumor weight among Sh-circXPO1 and sh-NC transfected groups (*n* = 5 for each group). **(C, D)** Changes in tumor volume during this period. Tumor size was determined as (length × width^2^)/2. **(E)** Subcutaneous tumor specimens were subjected to H&E staining and IHC staining of Ki-67 and DDIT4 in sh-NC and sh-circXPO1 groups. **(F)** The proportion of ki-67 positive cells between two groups. **(G)** Quantitative analysis of DDIT4 between two groups. *p < 0.05, **p < 0.01, ***p < 0.001. H&E, hematoxylin and eosin; IHC, immunohistochemistry; Sh, short hairpin.

To reveal the biology function of circXPO1 *in vivo*, MM.1S cells were transfected with Sh-circXPO1 or Sh-NC and injected under the skin of nude mice, with tumor volume being assessed every 2 days. Compared with Sh-NC transfected mice, the tumor growth rate of the Sh-circXPO1 transfected mice was significantly reduced, with tumor size less than the control group (Fig. 9A–D). By IHC, DDIT4 and ki-67 expression was lower in the experimental group than in the control group (Fig. 9E).

In addition, count analysis displayed that the proportion of ki-67 positive cells in the sh-circXPO1 group was lower than that in the sh-NC group (Fig. 9F). Quantitative analysis showed that DDIT4 expression was mostly positive in the sh-NC group and low positive or negative in the sh-circXPO1 group (Fig. 9G). This result was consistent with the *in vitro* results of DDIT4 expression that decreased after si-circXPO1 knockdown. These results demonstrated that the growth rate of MM cells in nude mice is significantly affected by the silencing of circXPO1.

## Discussion

MM is a hematological disease with a high and increasing incidence rate. Although treatment for MM has improved, a large number of patients still recrudesce or succumb within 2 years of treatment (Eschenhagen et al., 2017). At present, MM cannot be cured (Beltrami et al., 2003). Therefore, it is necessary to continue to investigate underlying pathological mechanisms as a means by which to establish relevant prognoses and treatment targets for better patient care.

CircRNAs comprised two components: an exon and/or intron that can intervene the expression of downstream gene by acting as a sponge for miRNAs (Panda, 2018). This form of regulation controls the proliferation and apoptosis of tumor cells and is associated with tumor pathogenesis (Ebbesen et al., 2016).

By high-throughput sequencing, circRNAs were differentially expressed in the bone marrow of MM patients and healthy controls. CircXPO1 was found to be elevating expression in newly diagnosed MM patients, with circXPO1 levels related to blood β_2_-MG and the ISS stage. Knockdown of circXPO1 in MM cells reduced cell proliferation, increased the apoptotic ratio, and arrested the cell cycle at G0/G1 phase. With overexpression of circXPO1, cell proliferation was increased, apoptosis was decreased, and the cell cycle progressed to the S phase, suggesting that circXPO1 acts as a carcinogenic agent in MM.

CircRNA usually acts as a sponge for the miRNA and binds to miRNA, preventing target genes of miRNA from binding to it to regulate gene expression (Panda, 2018). This study confirmed that circXPO1could bind to miR-495-3p. miR-495-3p was a tumor suppressor in tumorigenesis (Huldani et al., 2023). It has been reported to play a significant part in the pathology of several tumors.

For example, miR-495-3p was decreased in acute myeloid leukemia and inhibited the growth of leukemia cells (Rittavee et al., 2023). It has been confirmed that miR-495-3p down-regulates *HMGB1* expression to inhibit cell proliferation and migration in colorectal cancer (Zhang et al., 2022). However, miR-495-3p expression and function in MM have not been studied.

Our research herein for the first time revealed that miR-495-3p decreased expression, increased the level of miR-495-3p, inhibited MM cell proliferation and cell cycle progression, and promoted apoptosis. In addition, decreasing miR-495-3p rescued MM cell proliferation, cell cycle progression, and apoptosis after circXPO1 silencing, suggesting that circXPO1may promote MM development via regulating miR-495-3p.

Targets for circXPO1/miR-495-3p were investigated in the meantime. Dual luciferase assay verified that *DDIT4* was the target of miR-495-3p. *DDIT4* is associated with several cancers, including melanoma, leukemia, breast cancer, and lung cancer (Pinto et al., 2017). *DDIT4* was highly expressed in MM, and after knockdown prompted apoptosis of MM cells, impairing tumor migration and invasion ability (Yu et al., 2022). *DDIT4* overexpression inhibits mTORC1 activity and induces bortezomib resistance in MM patients (Decaux et al., 2010).

It increases miR-495 in breast cancer initiating cells, and it promotes oncogenesis by the down-regulation of *DDIT4* (Hwang-Verslues et al., 2011). The present study demonstrated a regulatory relationship between miR-495 and *DDIT4*. As such, this study identified a role for the circXPO1/miR-495-3p/*DDIT4* regulatory axis in the MM pathological process.

In conclusion, circXPO1 expression was up-regulated in the bone marrow of newly diagnosed MM patients. Further, circXPO1 was shown to promote the MM pathological process by acting as a sponge for miR-495-3p, increasing *DDIT4* expression. Taken together, these consequences suggested that circXPO1 may be a potential therapeutic target for the treatment of MM patients.

## Ethics Statement

The research scheme of this study was approved by the Ethics Committee of Xi'an Jiaotong University (Approval No. 2015186), and all the candidates had provided informed consent. Experiments on animals were authorized by the Medical Biology Research Ethics Committee (Approval No. 2022-1498).

## Supplementary Material

Supplemental data

Supplemental data

## Data Availability

The microarray data generated in this study is available in the Gene Expression Omnibus database repository (www.ncbi.nlm.nih.gov/geo, Accession No. GSE208782).
