# Supplementary material for: Circular RNA circXPO1 Promotes Multiple Myeloma Progression by Regulating miR-495-3p/DNA Damage-Induced Transcription 4 Axis
Source: DNA Cell Biol. 2024 Jan 12;43(1):39–55. doi: 10.1089/dna.2023.0288 (PMC10825292; doi:10.1089/dna.2023.0288)
Supplement: Supplemental data [file Suppl_TableS1.docx]

Supplementary Table 1 circRNAs that interact with star miRNAs in multiple myeloma

| circRNA | FC (abs) | Regulation | circRNA type | Gene Symbol | MRE1 | MRE2 | MRE3 | MRE4 | MRE5 |
| --- | --- | --- | --- | --- | --- | --- | --- | --- | --- |
| hsa_circRNA_100775 | 2.4353129 | up | exonic | MPPED2 | [hsa-miR-574-5p](circRNA_MREs/hsa-miR-574-5p_vs_hsa_circRNA_100775.pdf) | [hsa-miR-452-5p](circRNA_MREs/hsa-miR-452-5p_vs_hsa_circRNA_100775.pdf) | [hsa-miR-145-3p](circRNA_MREs/hsa-miR-145-3p_vs_hsa_circRNA_100775.pdf) | [hsa-miR-29b-1-5p](circRNA_MREs/hsa-miR-29b-1-5p_vs_hsa_circRNA_100775.pdf) | [hsa-miR-553](circRNA_MREs/hsa-miR-553_vs_hsa_circRNA_100775.pdf) |
| hsa_circRNA_100238 | 2.434156 | up | exonic | SSBP3 | [hsa-miR-29b-1-5p](circRNA_MREs/hsa-miR-29b-1-5p_vs_hsa_circRNA_100238.pdf) | [hsa-miR-661](circRNA_MREs/hsa-miR-661_vs_hsa_circRNA_100238.pdf) | [hsa-miR-874-5p](circRNA_MREs/hsa-miR-874-5p_vs_hsa_circRNA_100238.pdf) | [hsa-miR-29b-2-5p](circRNA_MREs/hsa-miR-29b-2-5p_vs_hsa_circRNA_100238.pdf) | [hsa-miR-185-3p](circRNA_MREs/hsa-miR-185-3p_vs_hsa_circRNA_100238.pdf) |
| hsa_circRNA_102735 | 2.2265169 | up | exonic | XPO1 | [hsa-miR-329-5p](circRNA_MREs/hsa-miR-329-5p_vs_hsa_circRNA_102735.pdf) | [hsa-miR-125a-5p](circRNA_MREs/hsa-miR-125a-5p_vs_hsa_circRNA_102735.pdf) | [hsa-miR-138-1-3p](circRNA_MREs/hsa-miR-138-1-3p_vs_hsa_circRNA_102735.pdf) | [hsa-miR-580-5p](circRNA_MREs/hsa-miR-580-5p_vs_hsa_circRNA_102735.pdf) | [hsa-miR-125b-5p](circRNA_MREs/hsa-miR-125b-5p_vs_hsa_circRNA_102735.pdf) |
| hsa_circRNA_104423 | 2.1761766 | up | exonic | CDK14 | [hsa-miR-301a-5p](circRNA_MREs/hsa-miR-301a-5p_vs_hsa_circRNA_104423.pdf) | [hsa-miR-371a-3p](circRNA_MREs/hsa-miR-371a-3p_vs_hsa_circRNA_104423.pdf) | [hsa-miR-29b-1-5p](circRNA_MREs/hsa-miR-29b-1-5p_vs_hsa_circRNA_104423.pdf) | [hsa-miR-221-5p](circRNA_MREs/hsa-miR-221-5p_vs_hsa_circRNA_104423.pdf) | [hsa-miR-372-3p](circRNA_MREs/hsa-miR-372-3p_vs_hsa_circRNA_104423.pdf) |
| hsa_circRNA_104974 | 2.1183751 | up | exonic | DHRSX | [hsa-miR-215-3p](circRNA_MREs/hsa-miR-215-3p_vs_hsa_circRNA_104974.pdf) | [hsa-miR-192-3p](circRNA_MREs/hsa-miR-192-3p_vs_hsa_circRNA_104974.pdf) | [hsa-miR-34c-3p](circRNA_MREs/hsa-miR-34c-3p_vs_hsa_circRNA_104974.pdf) | [hsa-miR-20b-3p](circRNA_MREs/hsa-miR-20b-3p_vs_hsa_circRNA_104974.pdf) | [hsa-miR-19b-1-5p](circRNA_MREs/hsa-miR-19b-1-5p_vs_hsa_circRNA_104974.pdf) |
| hsa_circRNA_400101 | 58.7063979 | down | intronic | RPL7A | [hsa-miR-18b-5p](circRNA_MREs\hsa-miR-18b-5p_vs_hsa_circRNA_400101.pdf) | [hsa-miR-18a-5p](circRNA_MREs\hsa-miR-18a-5p_vs_hsa_circRNA_400101.pdf) | [hsa-miR-452-3p](circRNA_MREs/hsa-miR-452-3p_vs_hsa_circRNA_400101.pdf) | [hsa-miR-639](circRNA_MREs/hsa-miR-639_vs_hsa_circRNA_400101.pdf) | [hsa-miR-670-3p](circRNA_MREs/hsa-miR-670-3p_vs_hsa_circRNA_400101.pdf) |
| hsa_circRNA_001264 | 49.0818117 | down | antisense | ST6GALNAC3 | [hsa-miR-18a-3p](circRNA_MREs\hsa-miR-363-5p_vs_hsa_circRNA_102312.pdf) | [hsa-miR-607](circRNA_MREs/hsa-miR-431-5p_vs_hsa_circRNA_102312.pdf) | [hsa-miR-632](circRNA_MREs/hsa-miR-568_vs_hsa_circRNA_102312.pdf) | [hsa-miR-654-3p](circRNA_MREs/hsa-miR-338-3p_vs_hsa_circRNA_102312.pdf) | [hsa-miR-10b-3p](circRNA_MREs/hsa-miR-527_vs_hsa_circRNA_102312.pdf) |
| hsa_circRNA_003907 | 4.2709709 | down | intronic | FARP1 | [hsa-miR-4496](circRNA_MREs\hsa-miR-503-5p_vs_hsa_circRNA_102408.pdf) | [hsa-miR-362-5p](circRNA_MREs/hsa-miR-103a-2-5p_vs_hsa_circRNA_102408.pdf) | [hsa-miR-6847-5p](circRNA_MREs/hsa-miR-660-3p_vs_hsa_circRNA_102408.pdf) | [hsa-miR-18a-3p](circRNA_MREs/hsa-miR-625-5p_vs_hsa_circRNA_102408.pdf) | [hsa-miR-3670](circRNA_MREs/hsa-miR-431-3p_vs_hsa_circRNA_102408.pdf) |
| hsa_circRNA_404449 | 3.7204189 | down | exonic | PINK1 | [hsa-miR-6512-3p](circRNA_MREs/hsa-miR-18a-3p_vs_hsa_circRNA_001264.pdf) | [hsa-miR-18a-3p](circRNA_MREs/hsa-miR-607_vs_hsa_circRNA_001264.pdf) | [hsa-miR-6772-3p](circRNA_MREs/hsa-miR-632_vs_hsa_circRNA_001264.pdf) | [hsa-miR-4308](circRNA_MREs/hsa-miR-654-3p_vs_hsa_circRNA_001264.pdf) | [hsa-miR-370-3p](circRNA_MREs/hsa-miR-10b-3p_vs_hsa_circRNA_001264.pdf) |
| hsa_circRNA_105013 | 3.2362269 | down | exonic | BRWD3 | [hsa-miR-141-5p](circRNA_MREs/hsa-miR-141-5p_vs_hsa_circRNA_105013.pdf) | [hsa-miR-486-5p](circRNA_MREs/hsa-miR-486-5p_vs_hsa_circRNA_105013.pdf) | [hsa-miR-578](circRNA_MREs/hsa-miR-578_vs_hsa_circRNA_105013.pdf) | [hsa-miR-135a-5p](circRNA_MREs/hsa-miR-135a-5p_vs_hsa_circRNA_105013.pdf) | [hsa-miR-135b-5p](circRNA_MREs/hsa-miR-135b-5p_vs_hsa_circRNA_105013.pdf) |
| hsa_circRNA_030509 | 2.7271968 | down | exonic | MYCBP2 | [hsa-miR-6881-3p](circRNA_MREs/hsa-miR-650_vs_hsa_circRNA_022920.pdf) | [hsa-miR-6781-3p](circRNA_MREs/hsa-miR-6718-5p_vs_hsa_circRNA_022920.pdf) | [hsa-miR-2682-3p](circRNA_MREs/hsa-miR-602_vs_hsa_circRNA_022920.pdf) | [hsa-miR-135b-5p](circRNA_MREs/hsa-miR-3612_vs_hsa_circRNA_022920.pdf) | [hsa-miR-4753-3p](circRNA_MREs/hsa-miR-6840-5p_vs_hsa_circRNA_022920.pdf) |
| hsa_circRNA_100219 | 2.6548428 | down | exonic | FAF1 | [hsa-miR-135b-3p](circRNA_MREs/hsa-miR-5586-5p_vs_hsa_circRNA_091419.pdf) | [hsa-miR-298](circRNA_MREs/hsa-miR-4453_vs_hsa_circRNA_091419.pdf) | [hsa-miR-485-3p](circRNA_MREs/hsa-miR-145-5p_vs_hsa_circRNA_091419.pdf) | [hsa-miR-182-5p](circRNA_MREs/hsa-miR-3916_vs_hsa_circRNA_091419.pdf) | [hsa-miR-593-5p](circRNA_MREs/hsa-miR-3613-5p_vs_hsa_circRNA_091419.pdf) |
| hsa_circRNA_101958 | 26.5877192 | down | exonic | UBE2G1 | [hsa-miR-433-3p](circRNA_MREs/hsa-miR-433-3p_vs_hsa_circRNA_101958.pdf) | [hsa-miR-29b-2-5p](circRNA_MREs/hsa-miR-29b-2-5p_vs_hsa_circRNA_101958.pdf) | [hsa-miR-125a-3p](circRNA_MREs/hsa-miR-125a-3p_vs_hsa_circRNA_101958.pdf) | [hsa-miR-346](circRNA_MREs/hsa-miR-346_vs_hsa_circRNA_101958.pdf) | [hsa-miR-135b-5p](circRNA_MREs/hsa-miR-135b-5p_vs_hsa_circRNA_101958.pdf) |
